# Case Report: Thymic Carcinoma Metastatic to Small Bowel

**DOI:** 10.4137/cmo.s824

**Published:** 2008-07-09

**Authors:** Boris Kobrinsky, Inessa Khaykis, Day Hill, Lydia Petrovic, Herman Yee, Anurag Chandra, David L. Diehl

**Affiliations:** Division of Medical Oncology, Division of Gastroenterology, Department of Medicine, Department of Radiation Oncology and Department of Pathology, NYU School of Medicine, New York, NY

## Introduction

Thymomas are the most common primary anterior mediastinal tumor and account for 15% to 20% of all mediastinal masses.(1, 3, 4) Although the majority of thymomas appear histologically benign, some may behave like invasive epithelial malignancies, invading local structures or metastasizing to distant organs.(2) However, extrathoracic direct invasion or distant metastasis are extremely rare and only a few cases have been reported previously. Even fewer cases of gastrointestinal tract invasion of thymomas have been documented in the English literature.(5, 6) This is the first clinical report of mediastinal thymic carcinoma metastatic to proximal small bowel.

## Case Report

**Figure 1:**
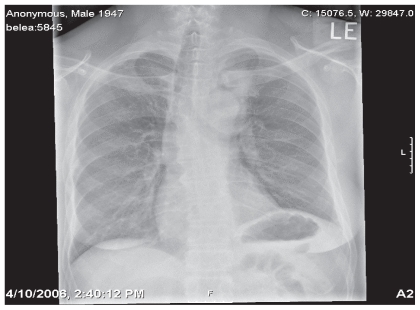
Chest X-ray showing superior mediastinal mass.

**Figure 2:**
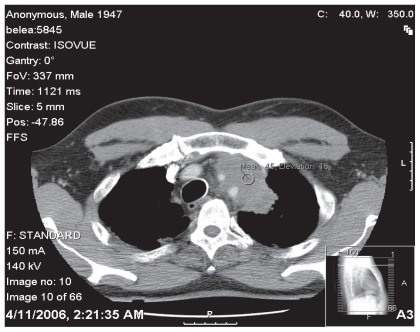
Chest CT demonstrating mass with involvement of aortic arch.

A 59 year old African American man with a 75 pack-year tobacco history presented with intermittent nocturnal anterior chest and non-productive cough. A chest x-ray showed a density adjacent to the aortic arch (Fig. 1). Chest CT revealed an 8 × 2.3 cm anterior mediastinal mass located superiorly which extended from the inner margin of the sternum along the aortic arch and encased the left subclavian and left common carotid arteries (Fig. 2).

**Figure 3:**
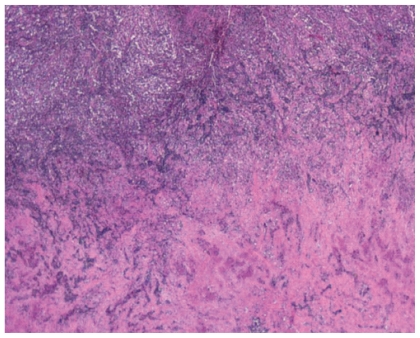
Hematoxylin-Eosin stain of primary tumor (40x).

**Figure 4:**
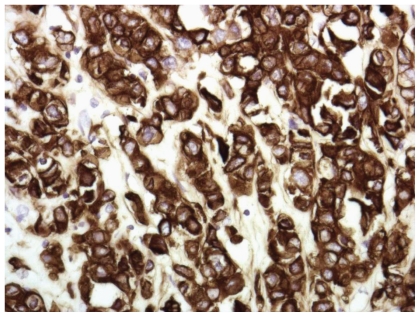
Immunohistochemistry staining with cytokeratine AE1/AE3.

**Figure 5:**
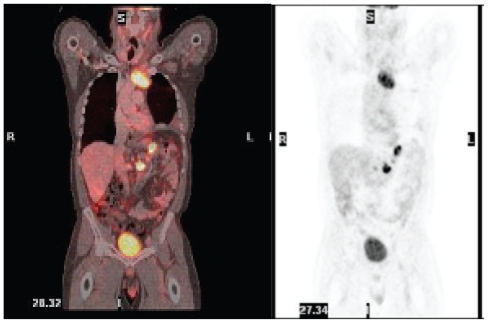
PET-CT demonstrates uptake in mediastinal mass, and two foci close to proximal small intestine.

**Figure 6:**
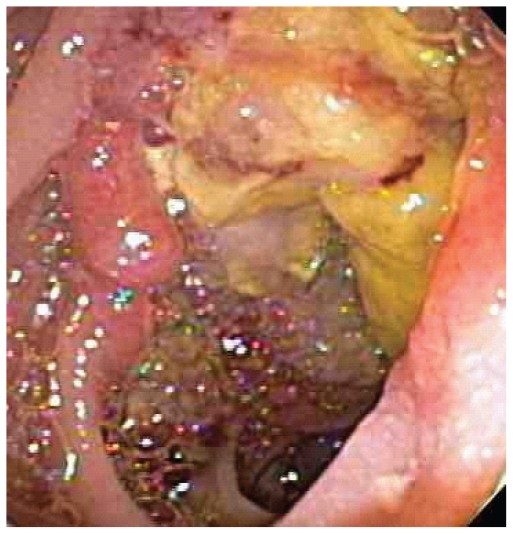
Endoscopic photograph of ulcerated mass in distal duodenum.

**Figure 7:**
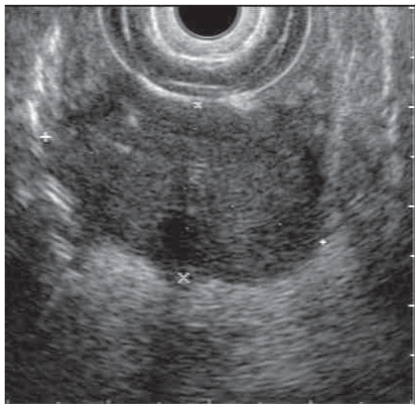
Endoscopic ultrasound of duodenal mass.

**Figure 8:**
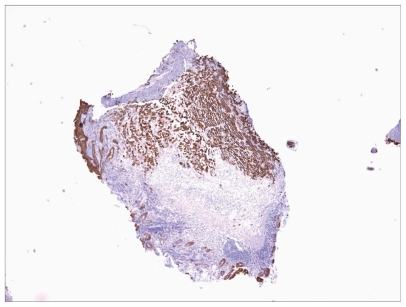
Biopsy of duodenal mass; immunohistochemistry positive for AE1/3 (epithelial marker).

He underwent mediastinoscopy with partial thoracotomy (Chamberlain procedure) and biopsy which showed poorly differentiated thymic carcinoma (Fig. 3) with immunohistochemistry positive for AE1/AE3, CD5, and vimentin (Fig. 4). PET scan showed the mediastinal mass with an SUV of 10. There was also uptake with SUV of 8.5 in a region close to the proximal intestine (Fig. 5). Because the PET suggested proximal bowel involvement, a colonoscope was used to visualize the proximal small bowel. This revealed a large fungating and ulcerated mass in the 4th (horizontal) part of the duodenum, about 3 cm in diameter (Fig. 6). EUS showed an ulcerated intramural lesion, involving deep mucosal layer, submucosa, muscularis propria and serosal (Fig. 7). Three malignant-appearing, round, hypoechoic lymph nodes with well-defined margins, measuring from 10 to 15 mm were visualized in the peritumoral region. The duodenal biopsy showed an ulcerated duodenal mucosa with an infiltrating epithelial neoplasm, composed of large polygonal cells, with abundant cytoplasm (Fig. 8). Only occasional intratumoral lymphocytes were present. The tumor did not exhibit any particular organoid or glandular growth pattern, and there was no tumor necrosis. A battery of immunostains was performed, including AE1/3, vimentin, CD3, CD20, CD10, S-100, monoclonal CEA, TTF, CDx-2, Ki-67, calretinin, and c-kit. The immunostains for AE1/3, mCEA, CK19, CK20 were strongly positive in tumor cells. CD10 was only focally weakly positive. All the other immunostains were negative.

The patient’s case was discussed in thoracic multi-disciplinary tumor board and the decision was made to treat with chemotherapy and radiation therapy. Radiation was added in this patient with metastatic disease for palliation as he had significant chest and left shoulder pain and also dyspnea on exertion. Given his age and no other significant medical co-morbid illnesses, we felt that he would tolerate concurrent treatment. The patient received 3 cycles of chemotherapy with cisplatinum and etoposide, both of which are reported to be active in thymic carcinoma.(18) Radiation was started with the first cycle for 15 fractions to a total dose of 4500 cGy. The targets were the primary 8 cm mediastinal thymic carcinoma and the chest wall lesion located anterior to the distal sternum. The radiation was delivered via parallel opposed antero-posterior (AP) and postero-anterior (PA) beams with 6 and 18 megavoltage (MV) photons respectively, to a dose of 2700 cGy. Additional dose of 1800 cGy was delivered to the primary mass via off-spinal-cord oblique fields with 6 MV photons. The chest wall lesion located anterior to the distal sternum was treated with 9MEV electrons to additional dose of 1800 cGy; thus, bringing the total dose to 4500 cGy. He did achieve significant pain relief at the end of his radiation course. His chemotherapy course was complicated by pulmonary embolism and febrile neutropenia. He had partial radiologic response to chemotherapy/radiation with decrease of his mediastinal mass to 6.6 × 2.8 cm. He felt very deconditioned with chemotherapy and declined further treatment after 3 cycles electing best supportive care.

## Discussion

The incidence of thymoma is 0.15 cases per 100,000 based upon data from the National Cancer Institute Surveillance, Epidemiology, and End Results (SEER) program.(11) Most patients are between 40 and 60 years of age, and there is slight male predominance. Up to one-half of thymomas may be found as an incidentally detected radiographic abnormality in an asymptomatic patient. In contrast, most patients with thymic carcinoma present with clinical symptoms, including cough, chest pain, phrenic nerve palsy, or superior vena cava syndrome.(12)

Although all thymomas are derived from the epithelial cells of the thymus, they represent a diverse group of tumors with varied histologic findings and biologic behavior. Histological subtypes include predominantly lymphocytic, predominantly epithelial, mixed lymphoepithelial, or spindle cell thymomas. The epithelial neoplastic cell usually grows slowly and lacks cytologic characteristics of malignancy. In contrast, thymic carcinomas, which account for less than 1 percent of thymic malignancies, have overtly malignant cytologic characteristics (e.g. anaplasia, cellular atypia, and increased proliferative activity) and capsular invasion.(2, 12)

Like thymoma, thymic carcinoma is most commonly located in the anterosuperior mediastinum. Approximately 80% of these patients have evidence of invasion of contiguous mediastinal structures, and 40% have radiologically apparent lymphadenopathy.(13) As with thymomas, local metastases often involve the pleura and mediastinal nodes, and sometimes, cervical and axillary lymph nodes, while distant metastases to the lungs, liver, brain and bone are rarely observed.(4, 7, 9) In addition renal and splenic metastasis have also been reported.(5, 6, 8)

Gastrointestinal tract invasion of thymomas is extremely uncommon. Brown et al. described a case of malignant thymoma with retroperitoneal extension below the diaphragm and invasion into the posterior wall of the stomach.(5) Fujikawa et al., reported a case of malignant thymoma that recurred three times during an 8-year period and invaded directly into the peritoneal cavity, involving the stomach, and transverse colon.(3) Bott-Kothari et al. reported a case of locally invasive mediastinal thymoma, which was initially treated with surgery and radiation therapy; 5 1/2 years later the patient was found to have a large mass in the abdomen and pelvis, involving the mesentery of the small intestine and a metastatic implant on the right ovary.(10)

Our case report is the first clinical case report of thymic carcinoma with distant metastatic disease involving small bowel at the time of diagnosis. Microscopic examination of the distal duodenal mass revealed the features of invasive poorly differentiated epithelial malignancy, the same subtype that was seen on the original mediastinal tumor biopsy. It has been reported that epithelial type thymomas are three times more likely to metastasize than as other types of thymoma.(9)

Non-invasive thymoma carries a generally good prognosis with 80% five-year survival rate. In contrast, the five-year survival rate of patients with primary thymic carcinoma varies from 34 to 65 percent(15) and was reported to be 35 percent at 10 years in one series.(16) Multimodality treatment with induction chemotherapy, surgery, and postoperative radiotherapy may offer improved survival in patients with unresectable malignant thymomas.(17)
